# An Unusual Cause of Lateral Knee Pain following Total Knee Replacement

**DOI:** 10.1155/2011/569413

**Published:** 2012-01-05

**Authors:** Lynne Barr, Vikas Khanduja, Julian Owen

**Affiliations:** Department of Orthopaedics and Trauma, Addenbrooke's Hospital, Cambridge University Hospitals, NHS Foundation Trust, Cambridge CB2 0QQ, UK

## Abstract

A 70-year-old male underwent elective total knee replacement for osteoarthritis. At initial review six weeks after surgery the prosthesis was functioning well and he was asymptomatic. He reattended clinic four months postoperatively having developed worsening pain on the lateral aspect of the knee but without any loss of function or stiffness of the joint. He subsequently underwent arthroscopy where synovial folds in the lateral gutter were debrided and entirely alleviated his symptoms. This is an unusual cause of pain following total knee replacement which has not been previously reported.

## 1. Introduction

Total knee replacement (TKR) is an increasingly common treatment for patients with arthritis. Over 70,000 such procedures are carried out in the United Kingdom each year. Pain following TKR may be due to various pathologies [1] including loosening, infection, component malposition, inadequate soft tissue balancing, arthrofibrosis [2], and soft tissue impingement. Diagnosis of the cause of pain therefore demands a thorough clinical evaluation and the use of appropriate investigations. Pain due to soft tissue impingement in the intercondylar notch [3] and patellofemoral joint [4, 5] has been previously reported. To our knowledge, symptoms arising from impingement in the lateral gutter have not been reported in the literature.

## 2. Case Presentation

A 70-year-old man presented to the orthopaedic clinic with an 18-month history of left knee pain, predominantly on the medial aspect of the joint. He experienced night pain, rest pain, and had increasing difficulty mobilising. He had undergone a successful right total knee replacement three years previously. Clinically his knee was in five degrees of fixed varus alignment, and range of movement was 0–100 degrees with pain at the limit of flexion. There was medial and lateral joint tenderness but no other significant findings on examination. Radiographs revealed severe degenerative changes in all three compartments.

**Figure 1:**
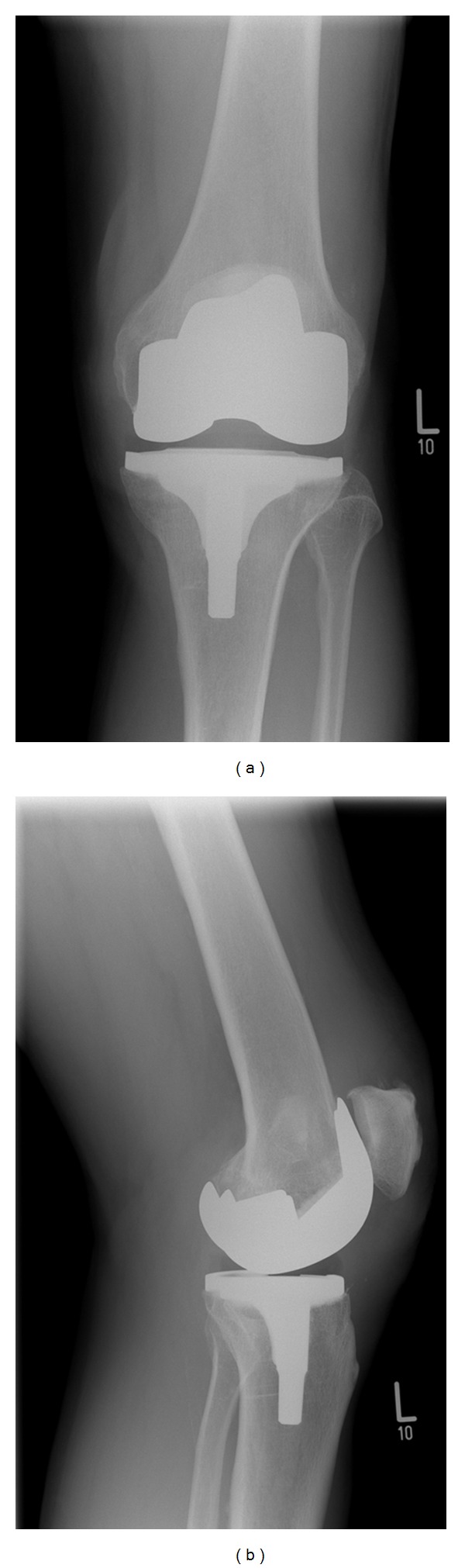
(a, b) Postoperative AP and lateral weight-bearing radiographs showing satisfactory alignment of the prosthesis.

**Figure 2:**
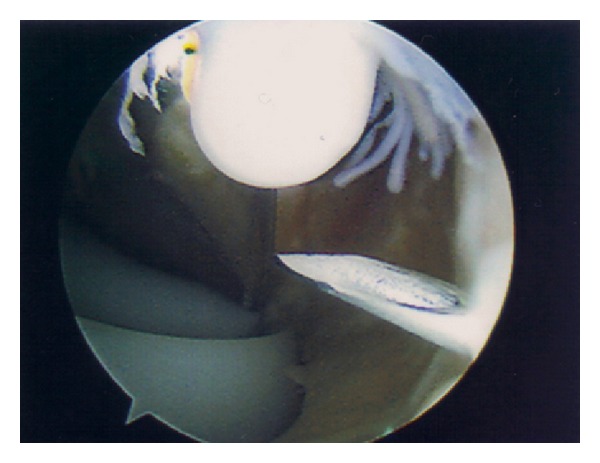
Arthroscopic view of lateral gutter showing fibrous tissue.

**Figure 3:**
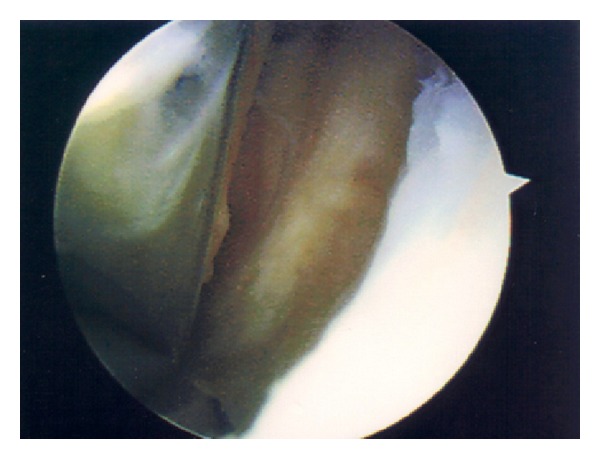
Arthroscopic view of lateral gutter following debridement.

Three months following his initial presentation, the patient underwent an uncomplicated cemented left total knee replacement with a Genesis II Posterior-Stabilised Prosthesis (Smith & Nephew). No synovial plicae were noted at surgery, nor was there any evidence of excess scar tissue within the joint. At review six weeks after surgery he was pain-free and walking unaided; range of movement was from 0 to 110 degrees flexion. At four months post-op he reattended clinic complaining of discomfort on the outer aspect of the left knee. This was treated with topical nonsteroidal anti-inflammatory drug (NSAID) gel and a course of physiotherapy. On review two months later, the pain had worsened such that his general practitioner had commenced amitriptyline as analgesia. He continued to complain of pain on the outside of the knee, particularly on weight-bearing, occasionally radiating down to the foot, and most severe on straightening the knee. On examination there was tenderness over the lateral aspect of the tibial plateau, but not around the fibular head or peroneal nerve. He had an unrestricted range of movement through 0–120 degrees. There was no motor or sensory deficit distally, and radiographs of the prosthesis were satisfactory (Figure 1). Inflammatory markers did not reveal any evidence of infection (CRP 1 and ESR 5). Due to the presence of a clear mechanical symptom of exacerbation of pain on knee extension, it was thought that there may be some soft tissue impingement in the lateral part of the joint. The patient underwent arthroscopy eight months following his initial surgery. On arthroscopic examination there were folds of fibrous tissue within the lateral gutter (Figure 2). These were debrided arthroscopically (Figure 3) and the knee lavaged. On review ten days following debridement the patient was entirely symptom-free. At latest review twelve months after arthroscopy, he remained pain-free, with range of movement 0–130 degrees, and had no functional limitations.

## 3. Discussion

Pain following knee arthroplasty can arise from a variety of causes including loosening, infection, component malposition, inadequate soft tissue balancing, arthrofibrosis, and soft tissue impingement. A thorough history and examination together with relevant radiological and laboratory investigations will identify most of the causes [1].

Arthrofibrosis has been reported in around 10% of patients following TKR [2] and is characterised by excessive scarring leading to painful stiffness of the joint. While arthrofibrosis causes a reduction in the range of movement of the joint, soft tissue impingement does not usually cause joint stiffness and is classically only symptomatic through a defined range of motion depending on where the impingement occurs. There have been previous reports of soft tissue impingement in the intercondylar notch [3] and impingement or tethering in the patellofemoral joint [4, 5] causing pain following TKR. Fabellar impingement is also a rare cause of such pain [6–9]. This case demonstrates a specific location and cause of pain due to soft tissue impingement following TKR which, to our knowledge, has not been previously reported.

Pain is the most common indication for proceeding to joint arthroplasty, and in the majority of cases surgery alleviates the patients' symptoms. Occasionally patients complain of postoperative pain which may or may not be the same as that which they experienced previously. Often there is no cause found for the pain, and generally it improves over time [10]. In a small group of patients the pain may be caused by impingement of scar tissues, and therefore surgical excision is likely to be curative. Identification of such patients requires careful and thorough clinical evaluation before the decision is made to proceed to surgery.

In the three cases of intercondylar notch soft tissue impingement reported by Hirsh and Sallis [3], all the patients developed pain of a differing nature to their preoperative pain 2–6 months following arthroplasty. Each complained of worsening pain on extension and also had a painful arc on resisted extension. The cases of soft tissue impingement and/or tethering within the patellofemoral joint reported by Pettine and Bryan [4] and Thorpe et al. [5] were similarly associated with an asymptomatic period of 5–24 months and 3–9 months, respectively. Each of these cases also described definite mechanical symptoms of pain during a particular phase of motion while not experiencing significant joint stiffness. In all the cases conservative management with analgesia and physiotherapy was unsuccessful. Similarly, in the current case the patient developed pain four months following joint replacement which differed both in site and nature from his preoperative pain. He experienced the most severe pain on extension of the knee with some relief on flexion. Conservative management failed to alleviate his symptoms, and therefore the decision was made to proceed to arthroscopy.

We would therefore suggest consideration of arthroscopic assessment of painful total knee replacements in those patients who have a delayed onset of new pain postoperatively, have clear mechanical symptoms not helped with conservative treatment, and in whom other possible diagnoses such as infection, loosening, malalignment, and instability have been ruled out.

## 4. Conclusion

Patients commonly report pain following total knee replacement. When the more common causes of pain have been ruled out, conservative measures have been unsuccessful, and the patient continues to experience pain of a mechanical nature, then soft tissue impingement must be considered as a possible aetiology, and arthroscopy should be considered to enable diagnosis and excision of the causative tissue.

##  Conflict of Interests

The authors declare that they have no conflict of interests.
